# Pregnancy outcomes in women with active anorexia nervosa: a systematic review

**DOI:** 10.1186/s40337-022-00551-8

**Published:** 2022-02-16

**Authors:** Jeremy Ryan Pan, Tina Yutong Li, Danny Tucker, Kai Yang Chen

**Affiliations:** 1 Townsville Hospital and Health Service, Townsville, QLD Australia; 2 College of Medicine and Dentistry, James Cook University, Townsville, QLD Australia

**Keywords:** Anorexia nervosa, Pregnancy, Pregnancy outcomes, Pregnancy complications, Maternal, Fetal, Neonatal

## Abstract

**Background:**

It is a common misconception that women with active anorexia nervosa (AN) are less likely to conceive. Pregnancies in women with AN are considered high risk. The purpose of this systematic review was to explore pregnancy complications in women with active AN, including maternal, fetal, and neonatal complications.

**Methods:**

The authors conducted a systematic review in accordance with PRISMA statement guidelines with stringent selection criteria to include studies on patients with active AN during pregnancy.

**Results:**

There were 21 studies included in our review. Anaemia, caesarean section, concurrent recreational substance use, intrauterine growth restriction, preterm birth, small-for-gestation (SGA) birth, and low birth weight were the most reported pregnancy complications in women with active AN, while the rates of gestational diabetes and postpartum haemorrhage were lower.

**Discussion:**

Women with active AN have a different profile of pregnancy complications comparing to malnourished women and women in starvation. We recommend early discussion with women diagnosed with AN regarding their fertility and pregnancy complications. We recommend clinicians to aim to improve physical and psychological symptoms of AN as well as correction of any nutritional deficiency ideally prior to conception. Management of pregnancies in women with active AN requires regular monitoring, active involvement of obstetricians and psychiatrist. Paediatric follow-up postpartum is recommended to ensure adequate feeding, wellbeing and general health of the infants. Psychiatric follow-up is recommended for mothers due to risk of worsening symptoms of AN during perinatal period.

**Supplementary Information:**

The online version contains supplementary material available at 10.1186/s40337-022-00551-8.

## Plain English summary

It is a common myth that women with active anorexia nervosa are less likely to become pregnant. Generally, pregnancies in women with active anorexia nervosa are considered high risk. This review looked at pregnancy complications in women with active anorexia nervosa that affect the mothers as well as unborn and newborn babies. There are number of complications reported, most commonly, anaemia, increased chance of birth by C-section, increased risk of substance use in mothers, poor growth of unborn babies, and smaller babies at birth. Mothers with anorexia nervosa are less likely to suffer from pregnancy related diabetes and postpartum haemorrhage. Interestingly, women with active anorexia nervosa tend to have different pregnancy complications comparing to women without adequate nutrition and women in starvation. It is important to mention that although women with anorexia nervosa carry higher risk of complications during pregnancy, the risk can be reduced with the help of obstetricians and psychiatrists. After birth, paediatrician visits can help identify any issue with the baby. Symptoms of anorexia nervosa could potentially get worse after delivering a baby. It is important to continue regular visits to a psychiatrist.

**Supplementary Information:**

The online version contains supplementary material available at 10.1186/s40337-022-00551-8.

## Background

Anorexia nervosa (AN) is a psychiatric disorder that affects approximately 1 in 100 women in the reproductive age group [1]. It is characterised by the inability to maintain normal weight most commonly due to intense fear of weight gain. While AN is classified as a psychiatric disorder, it often causes a range of physical consequences, such as electrolyte imbalance, amenorrhea secondary to hormonal derangements, impaired haematopoiesis, and decreased grey matter in the brain [2–5].

There is a common misconception that women with active AN cannot conceive because of irregular menses or amenorrhea [3]. In response to severely restricted energy intake, the hypothalamic-pituitary–gonadal axis (HPA) may produce hormonal secretion patterns similar to prepubertal or early pubertal individuals [5]. Luteinising hormone pulses in women with AN tend to have low pulse amplitude in general, which can result in amenorrhea [5]. AN has a fluctuating course of illness with periods of partial recovery and deterioration [6]. Some women with AN have irregular periods rather than amenorrhoea. This is likely due to fluctuating energy intake over time. During the partial recovery phase, briefly increased energy intake could reverse the HPA axis alteration and cause occasional ovulations that lead to pregnancy [5, 6]. Some studies estimated that there is no difference in fertility rates between women with AN and those without [7, 8]. The misconception about the fertility of women with active AN might explain a higher rate of unplanned pregnancy in women with AN. In a Norwegian study, up to 50% of pregnancies in women with AN were unplanned in comparison to 18.9% of the general population [9].

Pregnancies in women with active AN should be considered high risk. This is partially due to the strong link between maternal malnutrition and increased risk of adverse birth outcomes such as preterm delivery, low birth weight, maternal anaemia, and mortality [10, 11]. The pregnancy outcomes are highly dependent on the intake of adequate nutrients, such as complete proteins, lysine, omega-3 fatty acids, iron, and folate [12–14], which may be lacking in persons with active AN.

While nutritional status is an important predictor of pregnancy outcomes, it is not the sole contributor. Pregnant women with active AN could have a different pregnancy outcome profile in comparison to malnutrition due to additional psychological factors as well as other biological and social factors specific to AN [15–18]. For instance, perfectionism, low self-esteem, high level of dependency, and critical family environment are some of the factors implicated in the development of AN, which could cause increased maternal distress [17]. Maternal psychological stress has been associated with preterm delivery and antepartum complications, such as pre-eclampsia, threatened miscarriage, hyperemesis, and premature rupture of membranes [18]. Understanding of potential pregnancy complications in women with active AN would allow clinicians to make better decisions to avoid unfavourable outcomes.

To our knowledge, a comprehensive review of studies of pregnancy outcomes in women with active AN, with stringent inclusion criteria, has yet to be completed. There were some existing review articles investigating pregnancy outcomes in women that had been diagnosed with AN in the past without separate analysis for active AN during pregnancy [19–21]. This review aims to explore the primary research literature on pregnancy complications in women with active AN, including maternal, fetal, and neonatal complications.

## Methods

We searched PubMed, PsycINFO, CINAHL and SCOPUS using the MeSH terms: Anorexia Nervosa and Pregnancy, as well as keywords “pregnant”, “antenatal”, “perinatal”, “pregnancy”, “prenatal”, “gravidity”, and “gravida”. The searches were conducted on May 24^th^, 2021. This search was repeated before publication in August 2021 with no additional eligible study. We considered all published peer-reviewed articles written in English. Our review followed the Preferred Reporting Items for Systematic Reviews and Meta-Analyses (PRISMA) protocol. The methodology was published on PROSPERO.

### Inclusion criteria

Articles were included if they (1) contained primary research data including case studies and series that covers maternal, fetal, and/or neonatal outcomes (a neonate was defined to be less than 28 days of age), (2) studied patients that were pregnant with an active diagnosis of AN (defined as patients with clear ongoing AN symptoms or a diagnosis of AN with pre-pregnancy body mass index (BMI) of equal to or less than 18), and (3) studied patients were required to meet the International Classification of Diseases (ICD) or Diagnostic and Statistical Manual of Mental Disorders (DSM) criteria for anorexia nervosa.

### Exclusion criteria

Articles were excluded if not published in English or an English translation was not available.

### Data collection, synthesis, and article quality

We screened each entry by title, abstract, and then the full text to determine whether they met our inclusion criteria. In addition, we hand-searched the citations of relevant articles. This process was carried out independently by two researchers. When reviewers disagreed about an article’s inclusion status, a final decision was based on discussion. Data were extracted from all suitable articles regarding the study design, population studied, and pregnancy outcomes as outlined in Additional file 1: Appendix. Studies that included pregnancies other than singleton, such as twin pregnancies, were analysed separately. Article quality was analysed using the Mixed Methods Appraisal Tool (MMAT). The MMAT is a set of five, five-point checklists, each corresponding to a different study design [22]. We also utilised the National Health and Medical Research Council (NHMRC) level of evidence. Heavier weightage was given to articles with higher level of evidence (NHMRC). Pregnancy outcomes from selected articles were summarised based on their categories; maternal, fetal, and neonatal outcomes.

## Results

### Literature search

**Fig. 1:**
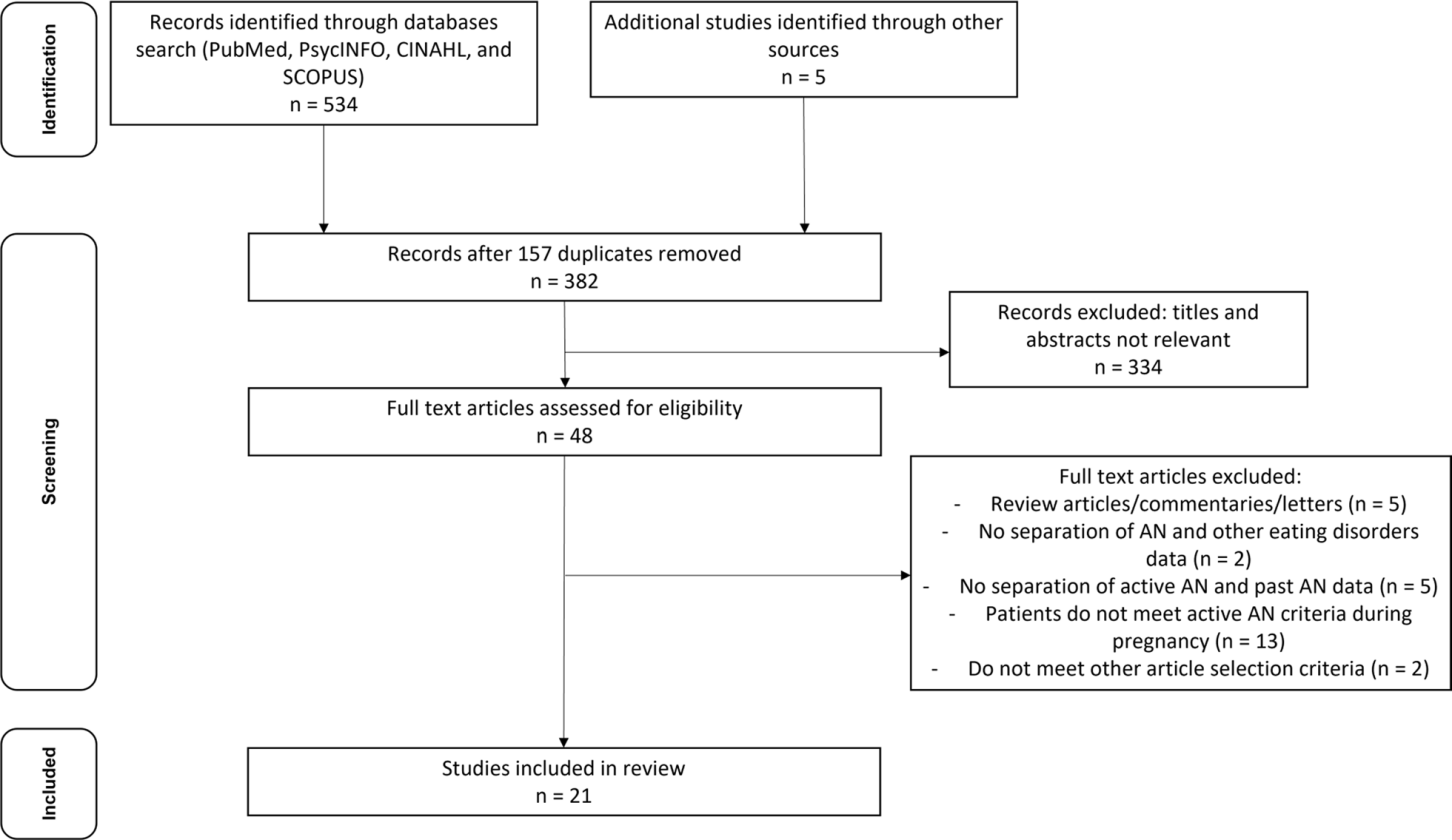
The Preferred Reporting Items for Systematic Reviews flowchart for the selection of studies

Our literature search yielded 534 articles. After deletion of duplicates and articles that did not meet our selection criteria, the remaining 21 studies were included in our review as outlined in Fig. 1. No eligible study was found through hand search. Among 21 studies included in this review, five were cohort studies, the remaining studies were case reports or series. Studies were originated in Europe (10), Asia (6), North America (4), and Oceania (1). The publication dates range from 1972 to 2020. Maternal age and parity varied between studies with the majority being between 20 to 30 years of age and primiparous. None of the studies in our review reported previous maternal complications prior to the onset of AN.

### Maternal outcomes

Seventeen studies mentioned maternal pregnancy outcomes (see Tables 1, 2, and 3). There were 22 reported maternal pregnancy complications that were more likely to occur in women with active AN. Mantel et al. compared the rate of maternal complications, such as hyperemesis and anaemia, between women with active and history of AN [23]. It was suggested that women with active AN were at higher risk of suffering from these pregnancy complications.

**Table 1:** Maternal outcomes women with active AN during pregnancies are more likely to occur in comparison to women without a history of AN—sorted by the number of studies and NHMRC level of evidence

Maternal outcomes	The number of studies and NHMRC level of evidence	Odds ratios (95% CI)
Anaemia	2 Retrospective cohort studies (Level III-2) [23, 24], 1 Case report (Level IV) [25]	2.1 (1.3–3.2) [23]
		2.39 (1.20–4.76) [24]
Caesarean section	1 Retrospective cohort study (Level III-2) [7], 3 Case reports (Level IV) [26, 27, 31]	–
Recreational substance use including smoking and alcohol use	1 Retrospective cohort study (Level III-2) [28], 1 Case series (Level IV) [29], 1 Case report (Level IV) [31]	–
Hyperemesis	1 Retrospective cohort study (Level III-2) [23], 1 Case series (Level IV) [33]	4.9 (3.5–6.9) [23]
Precipitous labour	1 Retrospective cohort study (Level III-2) [28]	1.43 (1.12–1.82) [28]
Acute liver failure	1 Retrospective cohort study (Level III-2) [28]	1.90 (1.12–3.21) [28]
Intensive care unit admission	1 Retrospective cohort study (Level III-2) [28]	1.86 (1.06–3.28) [28]
Premature contractions	1 Retrospective cohort study (Level III-2) [24]	2.31 (1.05–5.11) [24]
Worsening anorexia nervosa during perinatal period	2 Case reports (Level IV) [30, 31], 2 Case series (Level IV) [29, 32]	–
Femoral fractures secondary to osteoporosis	2 Case reports (Level IV) [26, 27]—one study was twin pregnancy [27]	–
Electrolyte imbalances	2 Case report (Level IV) [30, 34]	–
Hypoproteinemia	2 Case reports (Level IV) [34, 35]	–
Metabolic alkalosis	1 Case report (Level IV) [30]	–
Central diabetes insipidus	1 Case report (Level IV) [36]	–
Poor lactation	1 Case report (Level IV) [31]	–
Child neglect	1 Case report (Level IV) [31]	–
Symphysis pubis dysfunction	1 Case series (Level IV) [33]	–
Severe and early Braxton Hicks	1 Case series (Level IV) [33]	–
Refeeding syndrome	1 Case report (Level IV) [34]	–
Poor weight gain	1 Case report (Level IV) [37]	–
Thrombocytopenia	1 Case report (Level IV) [25]	–
Leukopenia	1 Case report (Level IV) [25]	–

**Table 2:** Maternal outcomes women with active AN during pregnancies are more likely to occur in comparison to women without a history of AN—sorted by categories: medical, surgical, obstetric, psychiatric, and others

Categories	Maternal outcomes	Odds ratios (95% CI)
Medical	Anaemia [23–25]	2.1 (1.3–3.2) [23]
		2.39 (1.20–4.76) [24]
	Thrombocytopenia [25]	–
	Leukopenia [25]	–
	Electrolyte imbalances [30, 34]	–
	Metabolic Alkalosis [30]	–
	Acute liver failure [28]	1.90 (1.12–3.21) [28]
	Hypoproteinemia [34, 35]	–
	Hyperemesis [23, 33]	4.9 (3.5–6.9) [23]
	Central Diabetes Insipidus [36]	–
	Refeeding syndrome [34]	–
Surgical	Femoral fractures secondary to osteoporosis [26, 27]	–
Obstetric	Caesarean section [7, 26, 27]	–
	Precipitous labour [28]	1.43 (1.12–1.82) [28]
	Premature contractions [24]	2.31 (1.05–5.11) [24]
	Symphysis pubis dysfunction [33]	–
	Severe and early Braxton Hicks [33]	–
	Poor weight gain [37]	–
Psychiatric	Recreational substance use [28, 29]	–
	Worsening anorexia nervosa during perinatal period [29–32]	–
Others	Intensive care unit admission [28]	1.86 (1.06–3.28) [28]
	Poor Lactation [31]	–
	Child neglect [31]	–

**Table 3:** Maternal outcomes women with active AN during pregnancies are less likely to occur in comparison to women without a history of AN—sorted by the number of studies and NHMRC level of evidence

Maternal outcomes	Frequency and NHMRC level of evidence	Odds ratios (95% CI)
Gestational diabetes	1 Retrospective cohort study (Level III-2) [28]	0.57 (0.44–0.73) [28]
Postpartum haemorrhage	1 Retrospective cohort study (Level III-2) [28]	0.76 (0.62–0.93) [28]
Behavioural symptoms related to disordered eating of AN but persisting cognitive symptoms	1 Case series (Level IV) [33]	–

Based on the level of evidence, anaemia [23–25], caesarean section [7, 26, 27] and concurrent recreational substance use [28, 29] were the most commonly reported by the studies included. In comparison to women without a history of AN, the odds ratios (OR) were 2.1 (95%CI 1.3–3.2) [23] and 2.39 (95%CI 1.20–4.76) [24] for anaemia.

When reporting rates of caesarean section, Bulik et al. reported 13% of pregnancies from women with active AN were delivered by a caesarean section compared to 6% of pregnancies in women without a history of AN [7]. They suggested that the statistical difference could not be determined due to the small sample size of the study. Linna et al. reported no statistically significant difference between elective caesarean rates among women with active AN and without a history of AN [24].

Ante et al. found the rate of substance use disorder among women with AN to be 2.7% in comparison to 1.5% in women without AN [28].

One study reported that postpartum haemorrhage [28] was less likely to occur in pregnancies of women with active AN. It was not specified whether the postpartum haemorrhage was primary or secondary. In comparison to women without a history of AN, the OR was 0.76 (95%CI 0.62–0.93) [28] for postpartum haemorrhage.

One study found no significant difference in rates of gestational diabetes [24], while another study found reduced risk in active AN with the OR of 0.57 (95%CI 0.44–0.73) [28].

Two case reports and two case series found that the behaviour symptoms (such as purging behaviour, restrictive eating and the use of laxatives) related to disordered eating in the woman with AN were worsened during pregnancy [29–32]. One case report found the opposite, where the patient had reduction of behaviour symptoms during pregnancy, but emotional and cognitive symptoms persisted [33].

### Fetal outcomes

Five studies reported fetal pregnancy outcomes (see Table 4). Varying degrees of intrauterine growth restriction accounts for the most reported adverse fetal outcome [24, 25, 30, 31, 38, 39]. Fetal distress was mentioned in one case report [30].

**Table 4:** Fetal outcomes women with active AN during pregnancies are more likely to occur in comparison to women without a history of AN—sorted by the number of studies and NHMRC level of evidence

Fetal outcomes	Frequency and NHMRC level of evidence	Odds ratios (95% CI)
Intrauterine growth restriction (IUGR)	1 Retrospective cohort study [24], 5 Case reports (Level IV) [25, 30, 31, 38, 39]	2.59 (1.43–4.71) [24]
Fetal distress	1 Case report (Level IV) [30]	–

### Neonatal outcomes

Seventeen studies mentioned neonatal pregnancy outcomes (see Tables 5 and 6). There were twelve reported neonatal complications that were more likely to occur in infants of women with active AN. A Swedish retrospective cohort study compared the rates of neonatal complications and suggested that neonates of women with active AN were at higher risk of preterm birth, small-for-gestation (SGA) birth, and microcephaly [23]. Hospitalisation due to AN during pregnancy was associated with a higher risk of adverse neonatal outcomes [28].

**Table 5:** Neonatal outcomes women with active AN during pregnancies are more likely to occur in comparison to women without a history of AN—sorted by the number of studies and NHMRC level of evidence

Neonatal outcomes	Frequency and NHMRC level of evidence	Odds ratios (95% CI)
SGA birth	3 Retrospective cohort study (Level III-2) [23, 24, 28], 1 Prospective Cohort Study (Level III-2) [40], 3 Case reports (Level IV) [30, 38, 39]	1.52 (1.35–1.72) [28]
		2.1 (1.3–3.6) [23]
		2.20 (1.23–3.93) [24]
		2.90 (1.98–4.26) [40]
Preterm birth (< 37 weeks)	3 Retrospective cohort studies (Level III-2) [23, 24, 28], 4 Case reports [26, 27, 34, 41]—three studies was twin pregnancy [27, 34, 41], 1 Case series (Level IV) [29]	Premature < 37 weeks: 1.32 (1.13–1.55) [28], 2.0 (1.4–2.9) [23]
		Very premature < 28 weeks: 2.0 (1.4–3.0) [23], 4.59 (1.25–16.87) [24]
Low birth weight	2 Retrospective cohort study (Level III-2) [24, 28], 3 Case reports (Level IV) [25, 27, 39]—one offspring in a twin pregnancy [27], 1 Case series (Level IV) [29]	1.69 (1.44–1.99) [28]
		2.16 (1.30–3.58) [24]
Perinatal death (stillbirth or death during the neonatal period)	2 Retrospective cohort study (Level III-2) [24, 28], 1 Case report (Level IV) [35]	1.99 (1.20–3.30) [28]
		4.06 (1.15–14.35) [24]
Neonatal Intensive care admission	1 Retrospective cohort study (Level III-2) [28], 1 Case report (Level IV) [30]	1.33 (1.09–1.62) [28]
Cardiovascular disorders	1 Retrospective cohort study (Level III-2) [28], 1 Case report (Level IV) [35]	1.27 (1.03–1.56) [28]
Respiratory disorders	1 Retrospective cohort study (Level III-2) [28]	1.16 (1.02–1.31) [28]
Microcephaly	1 Retrospective cohort study (Level III-2) [23]	2.8 (1.5–5.0) [23]
Metabolic alkalosis	1 Case report (Level IV) [30]	–
Electrolyte imbalances	1 Case report (Level IV) [30]	–
Growth restriction	1 Case series (Level IV) [29]	–
Micropolygyria	1 Case report (Level IV) [25]	–

**Table 6:** Neonatal outcomes women with active AN during pregnancies are more likely to occur in comparison to women without a history of AN—sorted by categories: growth or development related, medical, and others

Categories	Neonatal outcomes	Odds ratios (95% CI)
Growth or development related	SGA birth [23, 24, 28, 30, 38–40]	1.52 (1.35–1.72) [28]
		2.1 (1.3–3.6) [23]
		2.20 (1.23–3.93) [24]
		2.90 (1.98–4.26) [40]
	Low birth weight [24, 25, 27–29, 39]	1.69 (1.44–1.99) [28]
		2.16 (1.30–3.58) [24]
	Microcephaly [23]	2.8 (1.5–5.0) [23]
	Growth restriction [29]	–
	Micropolygria [25]	–
Medical	Cardiovascular disorders [28, 35]	1.27 (1.03–1.56) [28]
	Respiratory disorders [28]	1.16 (1.02–1.31) [28]
	Metabolic alkalosis [30]	–
	Electrolyte imbalances [30]	–
Others	Preterm birth (< 37 weeks) [23, 24, 26–29, 34, 41]	Premature < 37 weeks: 1.32 (1.13–1.55) [28], 2.0 (1.4–2.9) [23]
		Very premature < 28 weeks: 2.0 (1.4–3.0) [23], 4.59 (1.25–16.87) [24]
	Perinatal death (stillbirth or death during neonatal period) [24, 28, 35]	1.99 (1.20–3.30) [28]
		4.06 (1.15–14.35)[24]
	Neonatal Intensive care admission [28, 30]	1.33 (1.09–1.62) [28]

Based on the level of evidence, preterm birth [23, 24, 26–28, 34, 40, 41], SGA birth [23, 24, 28, 30, 38–40], and low birth weight [24, 25, 27–29, 39] were the most reported by the studies included. Three of the case reports that mentioned preterm birth were twin pregnancies [27, 34, 41]. Only one infant of a case report with twin pregnancy had low birth weight [27].

Recent hospitalisation due to AN was associated with a higher risk of preterm birth, low birth weight and SGA birth [28]. For example, OR for SGA was 1.83 (95% CI 1.35–2.49) among women that were hospitalised less than two years prior to pregnancy. This decreased to 1.52 (95%CI 1.14–2.04) if the hospitalisation was between two to four years prior, and further decreased to 1.47 (95%CI 1.27–1.70) if the hospitalisation was more than five years prior. A similar relationship between OR and the length of time since the last hospitalisation was also observed for low birth weight and preterm birth.

Neonates born from women with active AN were less likely to be large-for-gestation (LGA) (see Table 7) [24, 28]. One case study reported normal and healthy infant of a woman with active AN during pregnancy [42]. One case study reported a preterm birth of an infant, but they were of normal weight [41]. A case series by Treasure and Russell suggested that while five neonates of women with active AN during pregnancy were born SGA and underweight, accelerated growth was observed in all infants within the first few months after birth when adequate nutrition was provided [39].

**Table 7:** Neonatal outcomes women with active AN during pregnancies are less likely to occur in comparison to women without a history of AN—sorted by the number of studies and NHMRC level of evidence

Neonatal outcomes	Frequency and NHMRC level of evidence	Odds ratios (95% CI)
LGA birth	2 Retrospective cohort study (Level III-2) [24, 28]	0.13 (0.02–0.91) [24]
		0.66 (0.54–0.80) [28]

## Discussion

This is the first review of its kind to summarise the literature on pregnancy outcomes in women with active AN during pregnancy. The protocol was published on PROSPERO. Studies included in this review were geographically diverse as seven out of 21 studies were from outside of Europe and North America.

The major limitation of this review is the quality of the studies included. Only five out of 21 studies are cohort studies, while the remaining articles are case reports or series. Our inclusion criteria ensured that the participants in the studies had active symptoms of AN during their pregnancy. It is worth noting that the studies did not specify the duration of active AN during the course of pregnancy or whether participants had active AN at the time of conception. A significant part of the results could be attributed to the Canadian cohort study conducted by Ante et al. [28]. The Canadian cohort study has a large sample size. It considered confounders and investigated different types of pregnancy complications. There are some limitations to the Canadian study. It was conducted using a provincial hospital database for Quebec, Canada that might have incomplete patient health information. Patients were identified as having AN only if the disease severity warranted a hospital admission. This would have excluded patients without the need for a hospital admission. The study did not report on the reason for hospital admission nor the subtypes of AN.

Some of the findings of this review could be potentially due to other independent variables. For example, preterm birth, one of the most reported neonatal outcomes in infants of women with active AN, is associated with maternal depression [43]. As psychiatric co-morbidities are common among patients with AN, the results from this review should be still considered clinically relevant [44].

Caesarean section is one of the most reported maternal outcomes in pregnant women with active AN. The results are conflicting from different cohort studies [7, 24]. It is unclear from the studies whether the caesarean section performed were emergency or elective. Poor mental health is associated with preference for elective caesarean section according to a Norwegian study [45]. It has been reported that women with eating disorders have a lower level of general mental health wellbeing [46]. It would be reasonable to speculate that the increased percentage of caesarean section among women with active AN could be partially attributed to mental health [7].

Increased hyperemesis was mentioned by one cohort study and one case study [23, 33]. As pre-pregnancy rate of vomiting behaviour was not reported, it is uncertain if the finding of the studies could be attributed to purging behaviour from AN. Further research would be required to determine its significance.

Maternal ketoacidosis, hypoglycaemia, and hypoinsulinaemia are commonly reported in studies of starvation during pregnancy [47, 48]. None of these complications were found in our review. One possible explanation could be the chronic adaptation of the body to a malnourished state might have reduced the vulnerability of women with AN during pregnancy. Maternal anaemia, postpartum haemorrhage, and hypertensive disorders such as pre-eclampsia have been reported in studies of malnourished pregnant women [49]. Maternal anaemia is associated with iron deficiency [49]. Hypertensive disorders are associated with inadequate calcium intake [50]. Both nutritional deficiencies are likely to be common among women with active AN. While anaemia is among the most reported maternal complications in pregnant women with active AN, they are less likely to have postpartum haemorrhage [28]. There is no significant difference in rates of hypertensive disorders between women with active AN and without. The reason for the discrepancies is unclear. One possible explanation is that postpartum haemorrhage and hypertensive disorders are commonly associated with maternal obesity [51–53]. Women with active AN are underweight by definition, while someone that is malnourished could still be obese or of normal weight.

IUGR has been found to be associated with maternal undernutrition [49], which concurs with the most reported fetal complication in pregnancies of women with active AN. It is unclear whether IUGR in women with active AN is a result of nutritional undersupply, placental failure, or both. While it would be reasonable to assume that nutritional undersupply to be the main contributor to IUGR, placental failure should be considered. One of the major risk factors for placental failure is smoking [54], which can be a common maternal comorbidity among women with active AN [28, 29]. Preterm birth and low birth weight are commonly reported neonatal complications among both women with active AN or simply malnourished [49].

Preterm birth can be either iatrogenic or spontaneous. Mantel et al. reported a higher risk for iatrogenic preterm birth than spontaneous among women with active AN [23]. Neural tube defects are more likely to occur in infants of malnourished women due to folate deficiency [49], while our review did not find elevated risk among infants of women with active AN. The reason for this lack of significance among women with active AN is unclear.

Based on the findings of this review, it is reasonable to suggest that pregnant women with active AN carry a higher risk of complications than the general population. However, it is important to note that the risk is not absolute, and the risk could potentially be overestimated due to selection bias towards women with severe AN. A healthy pregnancy is possible if appropriate support and management are provided. We recommend early discussion with women diagnosed with AN regarding their fertility and potential complications during pregnancy. This would empower them with the knowledge to make an informed decision about active family planning if they do not wish to become pregnant. For women that wish to conceive, it is reasonable to assume that improvement on physical and psychological symptoms of AN as well as correction of any nutritional deficiency before conception would result in better pregnancy outcomes. We also recommend routine screening of eating disorders at the first contact with obstetric services [55]. If a woman has current or past eating disorders, prompt referral to psychiatry should be considered in discussion with the patient [55].

There is a lack of research in area of management of pregnancy in women with active AN. Some guidelines exist to manage the more common complications mentioned in our review [56]. It is important to note that the guidelines are not specific for women with active AN. For pregnant women with active AN, the risk of complications can be estimated by using the length of time since last hospital admission due to AN with a shorter period carrying higher risk [28], as well as body mass index and severity of behavioural symptoms of AN during pregnancy. Most reported maternal and fetal complications, such as anaemia, recreational substance use, and IUGR, can be monitored by utilising regular antenatal growth scans and maternal weight measurements, laboratory investigations, recreational substance screening, and psychiatric assessment of mental state throughout pregnancy in addition to standard obstetric care. Abnormalities on monitoring should be intervened where possible. If hyperemesis is present, it is important to differentiate between hyperemesis gravidarum and a purging symptom of AN as the management would differ. Active nutritional rehabilitation should be utilised to ensure adequate maternal weight gain during pregnancy as well as optimisation of any micronutrient deficiency, such as iron and calcium. In-patient management should only be considered after providing appropriate support and active nutritional rehabilitation in an outpatient setting failed to maintain a healthy weight during pregnancy or severe abnormality found on investigations.

To the best of our knowledge, there is no known evidence-based preventative strategy to manage the risk of preterm birth in women with active anorexia nervosa. However, in the general population, a transvaginal ultrasound scan can be utilised at twelve weeks and during mid-trimester morphology scan to assess cervical length to assess the risk of preterm birth. If the cervix is less or equal to 25 mm, vaginal progesterone can be utilised to reduce the risk of spontaneous preterm birth [57, 58]. If the cervix is less than 10 mm, cervical cerclage can be considered [59].

The rate of gestational weight gain was not reported in the cohort studies in our systematic review and was inconsistently reported among case studies. It is unclear what rate of gestational weight gain would be sufficient to sustain a healthy pregnancy in women with active AN.

Further research should be conducted in management of pregnancy in women with active AN to provide more evidence-based approach, especially monitoring targets for maternal weight gain and fetal growth, management and prevention of pregnancy complications, and optimal model of care. Research in these key areas can potentially accelerate the development of comprehensive guidelines.

While there are many areas of uncertainty in the management of pregnancy in women with active AN, it is clear that there are many disciplines involved. It is important to have a clinician as the single point of contact for a patient, who can develop a care plan in collaboration with the patient and liaise with other specialties [55]. This can ensure continuity of care and ongoing support.

During the postpartum period, a multidisciplinary approach with a single point of contact and coordination should continue [55]. More frequent midwifery follow-up could be beneficial in early recognition of any maternal complications, general wellbeing of the mother and infants, and ensuring adequate feeding of infants. Regular paediatric follow-up should be arranged to ensure adequate growth of infants as well as their general health. Regular psychiatric follow-up for mothers after delivery is crucial as symptoms of AN could worsen during the perinatal period [29–32]. Social workers can be utilised for patients with complex social issues that can exacerbate AN. A detailed management protocol is out of the scope for this review. Further higher quality studies would be required to optimise the outcome of pregnancies in women with active AN.

## Conclusions

It is a common misconception that women with active AN cannot conceive due to disruption of the menstrual cycle. Pregnancy in women with active AN can be complicated by potential risks to the mother and fetus. Pregnancy outcomes in women with active AN differ from malnutrition and starvation. Aetiology of AN is multifactorial and this reflects on the range of associated complications requiring the involvement of multiple health disciplines for optimal obstetric care. Further higher quality studies would be required for the formulation of a detailed management protocol for pregnancy in women with active AN.

## Supplementary Information

**Additional file 1. Appendix 1:** Summary of article data**.**

## Data Availability

The data used to support the findings of this study are included within the article.

## Abbreviations

AN: Anorexia nervosa
IUGR: Intrauterine growth restriction
SGA: Small-for-gestational-age
LGA: Large-for-gestational-age
