# Supplementary material for: Pregnancy outcomes in women with active anorexia nervosa: a systematic review
Source: J Eat Disord. 2022 Feb 16;10:25. doi: 10.1186/s40337-022-00551-8 (PMC8848585; doi:10.1186/s40337-022-00551-8)
Supplement: Supplementary file 1 — Additional file 1. Appendix 1: Summary of article data. [file 40337_2022_551_MOESM1_ESM.docx]

Appendix 1. Summary of Article Data

| **Author and Year** | **Title** | **Design (NHMRC level of evidence)** | **Sample** | **Exposure** | **Control** | **Results** | **MMAT** |
| --- | --- | --- | --- | --- | --- | --- | --- |
| Ante et al. 2020 [23] | Pregnancy outcomes in women with anorexia nervosa | Retrospective Cohort Study (Level III-2) | 2,134,945 singleton pregnancies, 1,910 of which were in women with AN in Quebec, Canada from 1989 to 2016  Demographic:   - Age: >80% aged 20-34 - Gender: Female - Race: Not recorded (NR) - Ethnicity: NR - Socioeconomic status: Average | Active AN | No history of AN | Maternal: increased risk of substance use disorders (2.7% versus 1.5%), precipitous labour (OR 1.43), acute liver failure (OR 1.9), intensive care unit admission (OR 1.86). Lowered risk of postpartum haemorrhage (OR 0.76), and gestational diabetes (OR 0.57).  Neonatal: increased risk of cardiovascular disorders (OR 1.27), respiratory disorders (OR 1.16). If hospitalised for anorexia nervosa, increased risk of stillbirth (OR 1.99), preterm birth (OR 1.32), low birth weight (OR 1.69), small-for-gestational-age (SGA) birth (OR 1.52), neonatal intensive care unit (NICU) admission (OR 1.33), and lowered risk of large-for-gestational-age (LGA) birth (OR 0.66).  More recent hospitalisation (preterm birth risk OR 1.92 for under 2 years prior, OR 1.37 for 2-4 years prior, OR 1.21 for over 5 years prior. Similar risk pattern for low birth weight and SGA birth). Hospitalisation during pregnancy due to AN (OR 3.0 for SGA. Similar for low birth weight.) is associated with a higher risk of adverse neonatal outcomes. Number of hospitalisations not correlated to more frequent/severe outcomes. | 3.1 Yes  3.2 Yes  3.3 Yes  3.4 Yes  3.5 Yes |
| Babatseva et al. 2020 [24] | A neonate with intrauterine growth restriction and pseudo-Bartter syndrome due to severe maternal eating disorder: A case report | Case Report (Level IV) | A primigravida 26-year-old mother with history of depression and anorexia nervosa since age of 18.  Demographic:   - Age: 26 - Gender: Female - Race: NR - Ethnicity: NR - Socioeconomic status: NR | AN and depression | Nil Control | Maternal: metabolic alkalosis, hypokalaemia, hypochloremia, hyponatremia, and worsening anorexia nervosa during second and third trimester of pregnancy  Fetal: intrauterine growth restriction, caesarean section due to fetal distress with Apgar scores of 7 and 8 at 1 and 5 minutes.  Neonatal: term 37-week SGA birth, uneventful NICU admission, total parenteral nutrition since day 1, alkalemia and electrolyte disorders similar to maternal with normal kidneys (pseudo-Bartter). | 4.1 Yes  4.2 Yes  4.3 Yes  4.4 Yes  4.5 Can’t tell |
| Bulik et al. 1999 [7] | Fertility and reproduction in women with anorexia nervosa: a controlled study | Retrospective Cohort Study (Level III-2) | 30 births by women with active AN in Christchurch, New Zealand  Demographic:   - Age: Mean age 32.4 - Gender: Female - Race: NR - Ethnicity: NR - Socioeconomic status: NR | Active AN | No active AN | 13% of births of women with active AN were delivered by a caesarean section in comparison to 6% in women without active AN. However, statistical power was insufficient to detect a significant difference as over 500 births would be required to detect a difference with p value of less than 0.05. | 3.1 Yes  3.2 Yes  3.3 Yes  3.4 Can’t tell  3.5 Yes |
| Hayashida et al. 2011 [25] | Anorexia nervosa and diabetes insipidus in pregnancy | Case Report (Level IV) | A 26-year-old Japanese female with active AN during pregnancy  Demographic:   - Age: 26 - Gender: Female - Race: Asian - Ethnicity: Japanese - Socioeconomic status: NR | Active AN | Nil Control | Central Diabetes Insipidus | 4.1 Yes  4.2 Yes  4.3 Yes  4.4 Yes  4.5 Yes |
| Ho 1985 [26] | Anorexia nervosa in pregnancy | Case Report (Level IV) | A 20-year-old woman with active AN during pregnancy, who was admitted for in-patient management during part of her pregnancy  Demographic:   - Age: 20 - Gender: Female - Race: NR - Ethnicity: NR - Socioeconomic status: NR | Active AN being managed in-patient | Nil Control | Maternal: Heavy smoker. Poor lactation. Caesarean section due to prolonged second stage of labour. Brief increase in food intake post-delivery, but quickly deteriorated. Neglected her newborn.  Fetal: intrauterine growth retardation  Neonatal: Nil complication | 4.1 Yes  4.2 Yes  4.3 Yes  4.4 Yes  4.5 Can’t tell |
| Kasahara et al. 2017 [27] | Bilateral femoral neck fractures resulting from pregnancy-associated osteoporosis showed bone marrow edema on magnetic resonance imaging | Case Report (Level IV) | A 38-year-old Japanese woman with active AN during pregnancy with a history of smoking and alcohol abuse as well as family history of osteoporosis  Demographic:   - Age: 38 - Gender: Female - Race: Asian - Ethnicity: Japanese - Socioeconomic status: NR | Active AN with a history of smoking and alcohol abuse as well as family history of osteoporosis | Nil Control | Maternal: bilateral femoral neck fractures secondary to pregnancy-associated osteoporosis, caesarean section delivery at 36 weeks  Neonatal: healthy and normal weight. No other information available. | 4.1 Yes  4.2 Yes  4.3 Yes  4.4 Yes  4.5 Can’t tell |
| Kasahara et al. 2018 [28] | Subchondral Insufficiency Fracture of the Femoral Head in a Pregnant Woman with Pre-existing Anorexia Nervosa | Case Report (Level IV) | A 40-year-old Japanese woman with active AN during twin pregnancy with a history of smoking  Demographic:   - Age: 40 - Gender: Female - Race: Asian - Ethnicity: Japanese - Socioeconomic status: NR | Active AN with a history of smoking | Nil Control | Maternal: left femoral neck fracture secondary to pregnancy-associated osteoporosis, caesarean section delivery at 36 weeks  Neonatal: twin delivered with weight of 2,630g and 1,1918g respectively | 4.1 Yes  4.2 Yes  4.3 Yes  4.4 Yes  4.5 Can’t tell |
| Kasahara et al. 2020 [29] | Smoking during Pregnancy Is a Predictor of Poor Perinatal Outcomes in Maternal Anorexia Nervosa: A Case Series and Single-Center Cross-Sectional Study in Japan | Case Series (Level IV) | 9 pregnancies by Japanese women with active AN who had a singleton pregnancy  Demographic:   - Age: Mean age 31 - Gender: Female - Race: Asian - Ethnicity: Japanese - Socioeconomic status: NR | Active AN with singleton pregnancy | No history of AN | Maternal: increased risk of worsening symptoms of AN during pregnancy. Higher smoking rate before and during pregnancy.  Neonatal: increased risk of premature birth, symmetric growth restriction (low birth weight and head circumference) | 4.1 Yes  4.2 Yes  4.3 Yes  4.4 Yes  4.5 Yes |
| Lakoff & Feldman 1972 [30] | Anorexia Nervosa Associated with Pregnancy | Case Report (Level IV) | A 26-year-old female with active AN during pregnancy who was medically managed as an inpatient with intravenous nutrition  Demographic:   - Age: 26 - Gender: Female - Race: Caucasian - Ethnicity: NR - Socioeconomic status: NR | Active AN during pregnancy with intravenous nutrition | Nil Control | Maternal: nil complication identified other than failure to gain weight prior to admission. Term delivery at 40 weeks.  Fetal: intrauterine growth retardation  Neonatal: small-for-gestational-age (SGA) at birth with APGAR score of 9 at 5 minutes. | 4.1 Yes  4.2 Yes  4.3 Yes  4.4 Yes  4.5 Can’t tell |
| Linna et al. 2014 [31] | Pregnancy, obstetric, and perinatal health outcomes in eating disorders | Retrospective Cohort Study (Level III-2) | 302 births from 182 mothers with AN in Finland  Demographic:   - Age: Mean age 29.4 - Gender: Female - Race: NR - Ethnicity: NR - Socioeconomic status: NR | Assumed active AN during pregnancy | No history of AN | Maternal: Increased risk of anaemia (OR 2.39), premature contractions (OR 2.31). Shorter first stage of labour (733 minutes on average vs 811 min on average for unexposed women). Statistically insignificant difference in terms of rate of induction of labour or elective caesarean section.  Fetal: Increased risk of intrauterine growth retardation (OR 2.59)  Neonatal: Increased risk of perinatal resuscitation (OR 1.06), perinatal death (OR 4.06), premature birth (OR 1.28) and very premature birth (OR 4.59), low birth weight (OR 2.16) and SGA (OR 2.20). Lower risk of LGA (OR 0.13) | 3.1 Yes  3.2 Yes  3.3 Yes  3.4 Yes  3.5 Yes |
| Madsen et al. 2009 [32] | Remission of eating disorder during pregnancy: five cases and brief clinical review | Case Report (Level IV) | A 24-year-old woman with active AN immediately prior to pregnancy and during early pregnancy  Demographic:   - Age: 24 - Gender: Female - Race: NR - Ethnicity: NR - Socioeconomic status: NR | Active AN immediately prior to pregnancy and during early pregnancy | Nil Control | Maternal: Patient was happy about being pregnant. Satisfactory weight gain and resolution of symptoms of AN by second trimester. Relapse of AN after 12 months post-partum. | 4.1 Yes  4.2 Yes  4.3 Yes  4.4 Yes  4.5 Can’t tell |
| Mantel et al. 2020 [33] | Association of Maternal Eating Disorders With Pregnancy and Neonatal Outcomes | Retrospective Cohort Study (Level III-2) | 279 women in Sweden with active AN prior to conception  Demographic:   - Age: Mean age 29.4 - Gender: Female - Race: NR - Ethnicity: NR - Socioeconomic status: NR | Active AN during pregnancy | Without history of AN | Maternal: Increased risk of hyperemesis (OR 4.9), anaemia (OR 2.1)  Neonatal: Increased risk of preterm birth (spontaneous and medically induced. OR 2.0), moderate preterm birth (OR 2.0), and very preterm birth (OR 2.0), SGA (OR 2.1), and microcephaly (OR 2.8), Apgar scores less than 7 at 5 min (OR 2.2).  Active AN vs Pre-existing AN: higher ORs across all complications | 3.1 Yes  3.2 Yes  3.3 Yes  3.4 Yes  3.5 Yes |
| Manzato et al. 2009 [34] | Pregnancy in severe anorexia nervosa | Case Report (Level IV) | A 19-year-old Arabian woman with active AN during pregnancy  Demographic:   - Age: 19 - Gender: Female - Race: Arabian - Ethnicity: NR - Socioeconomic status: NR | Active AN during prior and during pregnancy | Nil Control | Neonatal: preterm delivery at 36 weeks. Normal infant weight and Apgar scores. | 4.1 Yes  4.2 Yes  4.3 Yes  4.4 Yes  4.5 Can’t tell |
| Mason et al. 2012 [35] | The experience of pregnancy in women with a history of anorexia nervosa: An Interpretive Phenomenological Analysis | Case Series (Level IV) | 1 woman with AN relapse during pregnancy and 4 women with active AN during pregnancy  Demographic:   - Age: 23 - 35 - Gender: Female - Race: NR - Ethnicity: NR - Socioeconomic status: NR | Active AN during pregnancy | Nil Control | Maternal: Severe hyperemesis gravidarum, symphysis pubis dysfunction, and severe and early Braxton Hicks. Behavioural symptoms of AN decrease during pregnancy, but emotional/cognitive symptoms persist with the idea to return to formal AN behaviour postpartum | 4.1 Yes  4.2 Yes  4.3 Yes  4.4 Yes  4.5 Can’t tell |
| Micali et al. 2016 [36] | Size at birth and preterm birth in women with lifetime eating disorders: a prospective population-based study | Prospective Cohort Study (Level III-2) | 174 women in Denmark with active AN during pregnancy  Demographic:   - Age: Mean age 28.6 - Gender: Female - Race: NR - Ethnicity: NR - Socioeconomic status: Average | Active AN during pregnancy | Without AN | Neonatal: increased risk of SGA (OR 2.81), premature birth (OR 1.77) | 3.1 Yes  3.2 Yes  3.3 Yes  3.4 Yes  3.5 Yes |
| Milner & O’Leary 1988 [37] | Anorexia nervosa occurring in pregnancy | Case Report (Level IV) | A 23-year-old Caucasian female with active AN during pregnancy  Demographic:   - Age: 23 - Gender: Female - Race: Caucasian - Ethnicity: NR - Socioeconomic status: NR | Active AN during pregnancy | Nil Control | Maternal: hypoproteinemia  Neonatal: death after 9 days of age due to large foramen ovale and patent ductus arteriosus | 4.1 Yes  4.2 Yes  4.3 Yes  4.4 Yes  4.5 Can’t tell |
| Soyama et al. 2018 [38] | A case of refeeding syndrome in pregnancy with anorexia nervosa | Case Report (Level IV) | A 35-year-old Japanese woman with twin pregnancy and active AN (binge/purging subtype) during pregnancy managed as an in-patient  Demographic:   - Age: 35 - Gender: Female - Race: Asian - Ethnicity: Japanese - Socioeconomic status: NR | Active AN (binge/purging subtype) during pregnancy | Nil Control | Maternal: refeeding syndrome and associated electrolyte imbalance and hypoalbuminemia. Nil postpartum complication  Neonatal: preterm deliver at 36 weeks with both twins having normal weights and Apgar scores. | 4.1 Yes  4.2 Yes  4.3 Yes  4.4 Yes  4.5 Can’t tell |
| Stewart et al. 1987 [39] | Anorexia nervosa, bulimia, and pregnancy | Case Series (Level IV) | 4 women in Canada with active AN during pregnancy  Demographic:   - Age: 25 on average - Gender: Female - Race: NR - Ethnicity: NR - Socioeconomic status: NR | Active AN during pregnancy | AN or BN in remission | Maternal: lower weight gain  Neonatal: lower birth weight, but still within normal range | 4.1 Yes  4.2 Yes  4.3 Yes  4.4 Yes  4.5 Can’t tell |
| Strimling et al. 1984 [40] | Infant of a pregnancy complicated by anorexia nervosa | Case Report (Level IV) | A 35-year-old woman with active AN during pregnancy  Demographic:   - Age: 35 - Gender: Female - Race: NR - Ethnicity: NR - Socioeconomic status: NR | Active AN during pregnancy | Nil Control | Neonatal: Normal infant weighed 2,920g with normal Apgar score. Nil complication. Healthy weight gain throughout neonatal period on breastfeeding | 4.1 Yes  4.2 Yes  4.3 Yes  4.4 Yes  4.5 Can’t tell |
| Takei et al. 2012 [41] | Micropolygyria in an infant born to a patient with severe anorexia nervosa: a case report | Case Report (Level IV) | A 25-year-old Japanese woman with severe AN who underwent fertility treatment and became pregnant. Active AN during pregnancy  Demographic:   - Age: 25 - Gender: Female - Race: Asian - Ethnicity: Japanese - Socioeconomic status: NR | Active AN during pregnancy | Nil Control | Maternal: severe anaemia, thrombocytopenia, leukopenia  Fetal: SGA  Neonatal: low birth weight, micropolygyria | 4.1 Yes  4.2 Yes  4.3 Yes  4.4 Yes  4.5 Can’t tell |
| Treasure & Russell 1988 [42] | Intrauterine growth and neonatal weight gain in babies of women with anorexia nervosa | Case Report (Level IV) | 6 women with active AN during pregnancy  Demographic:   - Age: Mean age 31 - Gender: Female - Race: NR - Ethnicity: NR - Socioeconomic status: NR | Active AN during pregnancy | Normal population | Fetal: fetal growth diminished during the last trimester  Neonatal: abdominal circumference below the third centile at birth. Underweight at birth, but “catch-up” growth seen during neonatal period | 4.1 Yes  4.2 Yes  4.3 Yes  4.4 Yes  4.5 Can’t tell |
